# Finite-Time Disturbance Reconstruction and ADP-Assisted Attitude Regulation for Aerial-Refueling Receiver Aircraft

**DOI:** 10.3390/s26216814

**Published:** 2026-10-28

**Authors:** Yiming Guo, Wenbi Zhao, Bing Xiao

**Affiliations:** 1 School of Robotics and Embodied Intelligence, Chang’an University, Xi’an 710064, China; 2 School of Sciences, Xi’an Technological University, Xi’an 710021, China; zhaowenbi@xatu.edu.cn; 3 School of Automation, Northwestern Polytechnical University, Xi’an 710064, China; xiaobing@nwpu.edu.cn

**Keywords:** aerial refueling, receiver aircraft, adaptive dynamic programming, prescribed performance, finite-time disturbance observer

## Abstract

This paper investigates the attitude control problem of an aerial-refueling receiver aircraft subject to tanker-wake disturbances, atmospheric gusts, aerodynamic uncertainties, and inertia perturbations. To simultaneously improve transient performance, disturbance-rejection capability, and control efficiency, a prescribed-performance adaptive dynamic programming (ADP) framework is developed. First, a finite-time performance envelope is introduced to explicitly constrain the attitude response and guarantee that the prescribed performance boundary reaches its steady-state value within a finite time. Then, a high-precision finite-time disturbance observer, incorporating a finite-time differentiator, is constructed to reconstruct the lumped disturbance acting on the receiver aircraft. Based on the disturbance-compensated system, an ADP-based auxiliary policy is combined with a nonlinear damping term to coordinate transient regulation and control-effort optimization. Theoretical analysis establishes finite-time disturbance reconstruction and local uniform ultimate boundedness of the closed-loop signals under an explicitly stated input-to-state stability condition. Comparative simulations against a classical ADP controller and a robust finite-time controller demonstrate that the proposed method achieves smoother transient responses and a favorable balance between attitude regulation and control effort. Hardware-in-the-loop experiments on a three-axis turntable platform further verify the real-time implementability and effectiveness of the proposed control scheme.

## 1. Introduction

Aerial refueling is a key technology for extending aircraft endurance, increasing operational range, and enhancing mission flexibility [1,2]. During the refueling process, the receiver aircraft is required to maintain highly accurate motion while operating in close proximity to the tanker, which imposes stringent requirements on flight safety and control precision [3,4]. Reliable navigation and control also depend on continuous and accurate state information; recent multi-sensor UAV navigation architectures have emphasized resilient fusion of heterogeneous measurements to maintain robust positioning and navigation performance [5]. In particular, tanker-wake-induced aerodynamic interference, atmospheric gusts, aerodynamic uncertainties, and aircraft parameter variations may introduce significant multi-axis disturbances and degrade the attitude performance of the receiver aircraft [3,4,6]. Consequently, the development of an attitude controller capable of ensuring fast convergence, prescribed transient performance, effective disturbance rejection, and reasonable control effort is of considerable importance. Accordingly, this paper investigates prescribed-performance ADP attitude control for an aerial-refueling receiver aircraft subject to unknown lumped disturbances, with finite-time requirements imposed on the performance envelope and disturbance-reconstruction stage rather than on the complete state trajectory.

Conventional aircraft attitude control has extensively employed classical feedback and optimal control methods, such as proportional–integral–derivative (PID) control, linear quadratic regulation (LQR), and model predictive control (MPC) [7,8]. These approaches can provide satisfactory tracking performance under nominal operating conditions, but they generally do not explicitly reconstruct unknown external disturbances and lumped model uncertainties. To enhance disturbance-rejection capability, observer-based control strategies have therefore attracted considerable attention. Representative approaches include active disturbance rejection control (ADRC) based on extended state observers (ESOs) [9,10], as well as extended and sliding-mode disturbance observers [11,12]. Recent UAV studies have integrated disturbance estimation with robust nonlinear control. Bianchi et al. [13] employed high-order sliding-mode estimators to compensate for disturbances and parameter uncertainties in quadrotor control, while Thanh et al. [14] combined an extended state/disturbance observer with nonlinear robust backstepping for attitude stabilization and trajectory tracking. These studies demonstrate the effectiveness of observer-assisted robust control under uncertain operating conditions. Although adaptive observer designs have been developed to relax the requirement on prior disturbance information [11], many finite-time and high-order sliding-mode observer/differentiator schemes still rely on bounded disturbance derivatives, with their gain selection depending on known or conservatively estimated bounds [15,16]. Recent studies have advanced robust observer design for uncertain nonlinear systems with time-varying delays, including cascade observer architectures for UAVs carrying loads [17] and continuous–discrete high-gain observers for delayed nonlinear systems [18]. These studies provide valuable approaches to state estimation under complex dynamic conditions. For an aerial-refueling receiver aircraft, however, the lumped disturbance simultaneously contains tanker-wake effects, atmospheric gusts, aerodynamic uncertainties, and inertia perturbations. Consequently, an accurate disturbance bound is difficult to determine a priori. An excessively conservative bound may result in overly large observer gains and increased sensitivity to high-frequency effects, which is undesirable for high-precision attitude regulation. This motivates the development of a fast and high-precision finite-time disturbance observation scheme for the receiver aircraft.

Another important requirement for aerial refueling is the rapid stabilization of the receiver aircraft. During close-proximity flight, unexpected attitude deviations caused by wake vortices, gusts, and parameter variations should be suppressed within a short time to maintain a safe and accurate refueling configuration. Consequently, finite-time, fixed-time, and predefined-time control techniques have been increasingly introduced into receiver-aircraft control. Wu et al. developed a faster finite-time attitude stabilization method for receiver aircraft with practical uncertainties and disturbances [19], while predefined-time and fixed-time schemes were further investigated to provide more explicit convergence-time guarantees [20,21,22]. Related finite-time attitude-control studies on UAV platforms have also considered the coupled influence of external disturbances and actuator saturation [23]. Disturbance-robust finite-time attitude stabilization has also been investigated for multirotor UAVs using fast terminal sliding-mode control and disturbance-robust actuation [24]. Wang et al. extended this idea to a six-degree-of-freedom receiver model and proposed a predefined-time anti-disturbance control framework for the refueling phase [25]. Beyond convergence speed, transient behavior is also critical because large overshoots or attitude excursions may reduce the safety margin during close-proximity operation. Accordingly, recent studies have begun to combine finite/predefined-time convergence with prescribed-performance constraints. For example, Zhao et al. incorporated prescribed performance into a finite-time guidance and control framework for receiver-aircraft rendezvous [26], while Hu et al. developed a predefined-time prescribed performance scheme for receiver UAVs under actuator faults and constraints [27]. Nevertheless, in many existing fast-convergence designs, the main emphasis is placed on settling-time guarantees, disturbance rejection, and tracking-error constraints, whereas control effort is generally not treated as an explicit optimization objective. Since excessive control commands may lead to actuator saturation and deteriorate practical implementability, control-input expenditure should also be regarded as an important part of controller design. Recent studies explicitly considering actuator constraints and saturation compensation further confirm the importance of this issue [6,27]. Therefore, achieving rapid convergence and prescribed transient performance while simultaneously maintaining moderate control effort remains a meaningful problem for receiver-aircraft attitude control.

Optimal control provides a natural framework for explicitly balancing state regulation performance and control expenditure. However, for nonlinear aircraft dynamics, the associated Hamilton–Jacobi–Bellman (HJB) equation is generally difficult to solve analytically. Adaptive dynamic programming (ADP) and reinforcement learning therefore provide attractive alternatives by approximating the optimal value function and control policy online [28,29]. Their effectiveness has been demonstrated in various aerospace applications, including hypersonic vehicles, fixed-wing UAVs, morphing aircraft, and uncertain aircraft systems [30,31,32]. In particular, self-learning heuristic dynamic programming has been applied to air-vehicle flight control, while observer-assisted ADP designs have been used to address external disturbances and parameter uncertainties in aerial vehicles [33,34]. Recent studies have further incorporated prescribed performance, finite-time convergence, disturbance compensation, and actuator constraints into ADP-based control frameworks [35,36,37,38]. Nevertheless, these objectives are strongly coupled rather than independent. Faster convergence is often accompanied by stronger or nonsmooth control actions, which may increase control effort and aggravate actuator limitations. Meanwhile, prescribed-performance constraints impose additional requirements on transient responses, and the interaction between performance constraints, actuator saturation, and control-cost optimization has already been recognized as an important issue in optimal attitude control [37]. Finite-time prescribed-performance studies also show that convergence speed, transient constraints, and optimal regulation must be coordinated within a unified design framework [36]. Furthermore, when disturbance observers are embedded into ADP-based controllers, the disturbance reconstruction quality directly affects the compensated dynamics and thus influences both tracking and optimization performance [35,38]. Therefore, the key challenge is not simply to combine existing control techniques but to coordinate prescribed transient performance, rapid disturbance reconstruction, practical closed-loop regulation, and control-effort optimization within the same framework. This issue is particularly important for aerial-refueling receiver aircraft, where multi-source aerodynamic disturbances coexist with stringent attitude-performance requirements and limited control authority.

Compared with existing observer-based ADP schemes [33,34] and related prescribed-performance ADP approaches [35,36,37,38], the proposed framework integrates finite-time disturbance reconstruction, prescribed transient performance, and HJB-based auxiliary control optimization for aerial-refueling receiver aircraft. Specifically, the finite-time observer compensates for lumped disturbances, while the prescribed-performance transformation and critic-generated auxiliary policy coordinate transient regulation, disturbance rejection, and control-effort optimization. The main contributions are summarized as follows:

1. A high-precision finite-time disturbance observer is developed to reconstruct the multi-source lumped disturbance acting on the receiver aircraft, providing finite-time disturbance compensation for the control system.
2. A prescribed-performance ADP controller is constructed by combining a critic-generated auxiliary policy, disturbance compensation, and nonlinear damping. The analysis establishes bounded critic weights and local uniform ultimate boundedness under a stated input-to-state stability condition, without asserting finite-time convergence of the complete state vector.
3. Numerical simulations and HIL experiments are carried out, and comparative results with classical controllers verify the improved transient performance, disturbance-rejection capability, moderate control effort, and real-time implementability of the proposed method.

The remainder of this paper is organized as follows. Section 2 presents the receiver-aircraft dynamics and disturbance model. Section 3 develops the proposed prescribed-performance ADP control scheme and its boundedness analysis. Section 4 presents the simulation and HIL validation results. Section 5 concludes the paper.

## 2. Receiver Aircraft Dynamics in Aerial Refueling

**Figure 1:**
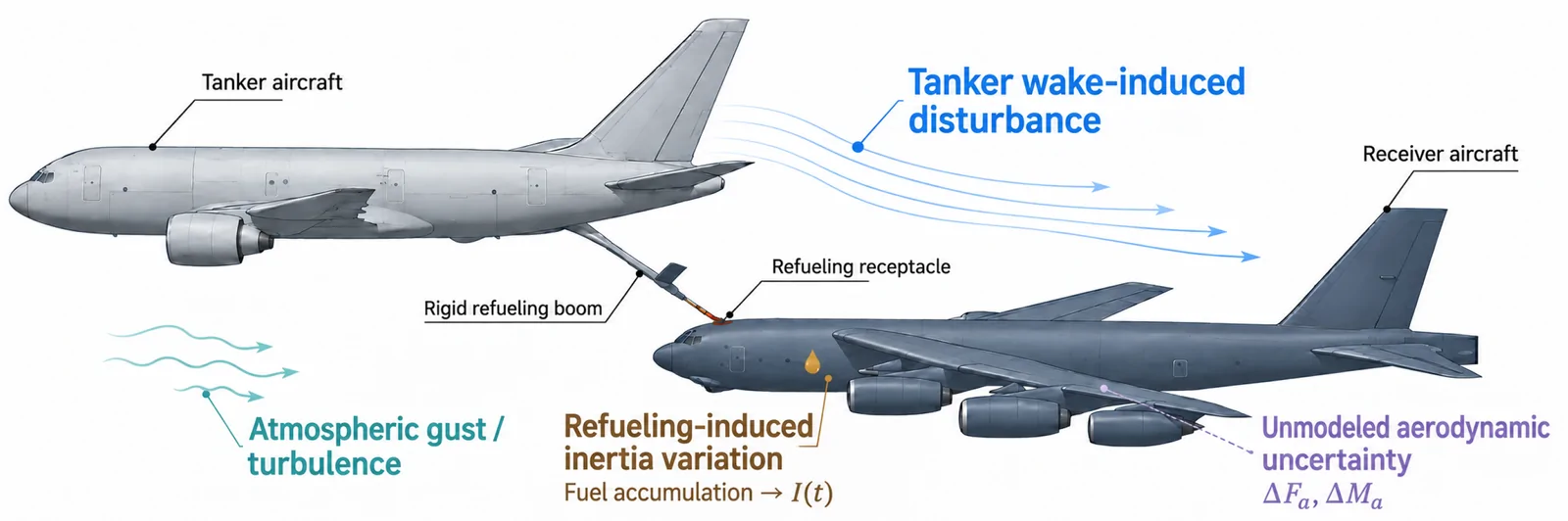
Main disturbances acting on the receiver aircraft during aerial refueling.

During aerial refueling, the receiver aircraft is affected by tanker wake, jet-wash, atmospheric gusts, and aerodynamic uncertainties, which may induce significant attitude disturbances. The main disturbance sources considered in this work are illustrated in Figure 1, including tanker-wake-induced moments, gust disturbances, aerodynamic uncertainties, and inertia perturbations. In this work, the receiver aircraft is modeled as a conventional fixed-wing aircraft. The focus is placed on attitude stabilization under tanker-induced aerodynamic disturbances, while the relative translational motion and docking geometry between the tanker and receiver are not explicitly considered. The geometry and reference parameters follow standard aerodynamic conventions.

Let $V={\left[v_{1}\;v_{2}\;v_{3}\right]}^{T}$ denote the linear velocity vector and $\omega ={\left[\omega_{1}\;\omega_{2}\;\omega_{3}\right]}^{T}$ the angular velocity vector expressed in the body frame. The translational and rotational dynamics of the receiver aircraft are governed by the Newton–Euler equations:

(1) $$\begin{array}{cc} m\dot{V} & =F_{\mathrm{aero}}+F_{\mathrm{thrust}}+F_{\mathrm{grav}}-\omega^{\times }mV, \end{array}$$

(2) $$\begin{array}{cc} J\dot{\omega } & =M_{\mathrm{aero}}+M_{\mathrm{thrust}}+M_{\mathrm{wake}}-\omega^{\times }J\omega , \end{array}$$

where *m* is the mass of the receiver aircraft and $J\in R^{3\times 3}$ is the inertia tensor. The terms $F_{\mathrm{aero}}$ and $M_{\mathrm{aero}}$ denote the nominal aerodynamic force and moment, respectively, $F_{\mathrm{thrust}}$ and $M_{\mathrm{thrust}}$ represent the propulsion force and moment, $F_{\mathrm{grav}}$ is the gravitational force, and $M_{\mathrm{wake}}$ denotes the additional aerodynamic moment induced by the tanker wake.

### 2.1. Aerodynamic Forces and Moments

The aerodynamic force in the body frame is expressed using the dynamic pressure $\bar{q}=\frac{1}{2}\rho {∥V∥}^{2}$, reference area $S_{\mathrm{ref}}$, and the body-to-wind transformation matrix $T_{\alpha \beta }$:

(3) $$F_{\mathrm{aero}}=\bar{q}S_{\mathrm{ref}}T_{\alpha \beta }\left[\begin{array}{c} -C_{D}\\ -C_{Y}\\ -C_{L} \end{array}\right],$$

where $C_{D}$, $C_{Y}$, and $C_{L}$ are the drag, side-force, and lift coefficients, respectively. The angle of attack α and sideslip angle β define the transformation $T_{\alpha \beta }$, which rotates the wind-axis force vector into the body frame.

The nominal aerodynamic moment is given by

(4) $$M_{\mathrm{aero}}=\bar{q}S_{\mathrm{ref}}\mathrm{diag}\left\{\bar{b},\bar{c},\bar{b}\right\}\left[\begin{array}{c} C_{l}\\ C_{m}\\ C_{n} \end{array}\right],$$

where $\bar{b}$ and $\bar{c}$ denote the wing span and mean aerodynamic chord, respectively, and $C_{l}$, $C_{m}$, and $C_{n}$ are the rolling, pitching, and yawing moment coefficients.

For the receiver aircraft, the aerodynamic coefficients are decomposed into static, angular-rate-dependent, and control-surface contributions. A local linear representation around the nominal aerial-refueling flight condition is adopted as

(5) $$\left[\begin{array}{c} C_{D}\\ C_{Y}\\ C_{L} \end{array}\right]=\left[\begin{array}{c} C_{D0}+C_{D\alpha }\alpha +C_{D\beta }\beta +C_{Dq}\frac{\bar{c}}{2V}q\\ C_{Y0}+C_{Y\beta }\beta +C_{Yp}\frac{\bar{b}}{2V}p+C_{Yr}\frac{\bar{b}}{2V}r\\ C_{L0}+C_{L\alpha }\alpha +C_{Lq}\frac{\bar{c}}{2V}q \end{array}\right]+\left[\begin{array}{ccc} 0 & 0 & 0\\ C_{Y\delta_{a}} & 0 & C_{Y\delta_{r}}\\ 0 & C_{L\delta_{e}} & 0 \end{array}\right]\left[\begin{array}{c} \delta_{a}\\ \delta_{e}\\ \delta_{r} \end{array}\right],$$

where p,q,r are the body-axis angular rates, V=∥V∥, and $\delta_{a},\delta_{e},\delta_{r}$ denote the aileron, elevator, and rudder deflections, respectively.

Similarly, the moment coefficients are modeled as

(6) $$\left[\begin{array}{c} C_{l}\\ C_{m}\\ C_{n} \end{array}\right]=\left[\begin{array}{c} C_{l0}+C_{l\beta }\beta +C_{lp}\frac{\bar{b}}{2V}p+C_{lr}\frac{\bar{b}}{2V}r\\ C_{m0}+C_{m\alpha }\alpha +C_{mq}\frac{\bar{c}}{2V}q\\ C_{n0}+C_{n\beta }\beta +C_{np}\frac{\bar{b}}{2V}p+C_{nr}\frac{\bar{b}}{2V}r \end{array}\right]+M_{\delta }\left[\begin{array}{c} \delta_{a}\\ \delta_{e}\\ \delta_{r} \end{array}\right],$$

where the control effectiveness matrix is

$$M_{\delta }=\left[\begin{array}{ccc} C_{l\delta_{a}} & 0 & C_{l\delta_{r}}\\ 0 & C_{m\delta_{e}} & 0\\ C_{n\delta_{a}} & 0 & C_{n\delta_{r}} \end{array}\right].$$

The aerodynamic coefficients used in this study follow the receiver-aircraft model adopted in our previous work [19], which is based on wind-tunnel test data. As this study focuses on control design and performance evaluation, the detailed aerodynamic coefficient curves are not presented here.

### 2.2. Gravity Force

The gravitational force expressed in the receiver-aircraft body frame is

(7) $$F_{\mathrm{grav}}=\left[\begin{array}{c} -\sin\theta \\ \sin\phi \cos\theta \\ \cos\phi \cos\theta \end{array}\right]mg,$$

where *g* is the gravitational acceleration.

### 2.3. Thrust Force and Moment

For a receiver aircraft with a propulsion system approximately aligned with the longitudinal body axis, the thrust vector is modeled as

(8) $$F_{\mathrm{thrust}}=\left[\begin{array}{c} T\\ 0\\ 0 \end{array}\right],$$

where T≥0 denotes the total thrust magnitude. The thrust-induced moment is assumed to be negligible compared with the aerodynamic and wake-induced moments, i.e., $M_{\mathrm{thrust}}\approx 0$.

### 2.4. Control Surface Moment Mapping

The control-surface-induced aerodynamic moment is extracted from the nominal aerodynamic moment model and regarded as the virtual control input for receiver-aircraft attitude regulation:

(9) $$\tau =\bar{q}S_{\mathrm{ref}}\mathrm{diag}\left\{\bar{b},\bar{c},\bar{b}\right\}M_{\delta }\delta ,$$

where

$$\delta ={\left[\delta_{a},\delta_{e},\delta_{r}\right]}^{T}.$$

For the subsequent attitude controller design, the generalized aerodynamic moment τ is used directly as the virtual control input. The actuator allocation from τ to the individual control-surface deflections is not explicitly considered. The commanded torque is assumed to be realizable within the admissible control-surface range, while the deflection constraints $|\delta_{i}|\;\leq \;\delta_{\max}$ and control-surface deflection-rate limits are not explicitly modeled.

### 2.5. Tanker-Wake-Induced Disturbance

During aerial refueling, the receiver aircraft may enter the nonuniform velocity field generated by the tanker wake. The resulting aerodynamic interference produces additional rolling, pitching, and yawing moments, which are represented as

(10) $$\tau_{\mathrm{wake}}=\left[\begin{array}{c} L_{\mathrm{wake}}\\ M_{\mathrm{wake}}\\ N_{\mathrm{wake}} \end{array}\right].$$

In addition to tanker-wake effects, the receiver aircraft is also subjected to atmospheric gusts and aerodynamic-model uncertainties. Therefore, the total disturbance torque is expressed as

(11) $$\tau_{d}=\tau_{\mathrm{wake}}+\tau_{\mathrm{gust}}+\bar{q}S_{\mathrm{ref}}\mathrm{diag}\left\{\bar{b},\bar{c},\bar{b}\right\}\left[\begin{array}{c} \Delta C_{l}\\ \Delta C_{m}\\ \Delta C_{n} \end{array}\right],$$

where $\tau_{\mathrm{gust}}$ denotes the moment disturbance caused by atmospheric turbulence, while $\Delta C_{l}$, $\Delta C_{m}$, and $\Delta C_{n}$ represent unmodeled aerodynamic variations excluding the nominal control effectiveness.

The tanker-wake moment is not assumed to be precisely known in the controller design. Instead, it is incorporated into the lumped disturbance and is reconstructed online by the proposed high-precision finite-time disturbance observer.

### 2.6. Attitude Kinematics and Control-Oriented Formulation

Using the Euler-angle representation $\Theta ={\left[\phi ,\theta ,\psi \right]}^{T}$, the attitude kinematics are described by

(12) $$\dot{\Theta }=\Pi \left(\Theta \right)\omega ,$$

where

$$\Pi \left(\Theta \right)=\left[\begin{array}{ccc} 1 & \sin\phi \tan\theta & \cos\phi \tan\theta \\ 0 & \cos\phi & -\sin\phi \\ 0 & \sin\phi \sec\theta & \cos\phi \sec\theta \end{array}\right].$$

Considering the inertia uncertainty of the receiver aircraft, the control-oriented attitude dynamics can be written as

(13) $$\left\{\begin{array}{c} \dot{\Theta }=\Pi \left(\Theta \right)\omega ,\\ \left(J_{0}+\Delta J\right)\dot{\omega }=-\omega^{\times }\left(J_{0}+\Delta J\right)\omega +\tau +\tau_{d}, \end{array}\right.$$

where $J_{0}$ denotes the nominal inertia matrix, ΔJ represents the inertia variation, and $\tau_{d}$ contains the tanker-wake disturbance, atmospheric gust disturbance, and unmodeled aerodynamic effects.

By isolating the perturbation associated with ΔJ, the above system can be reformulated as

(14) $$\left\{\begin{array}{c} \dot{\Theta }=\Pi \left(\Theta \right)\omega ,\\ J_{0}\dot{\omega }=-\omega^{\times }J_{0}\omega +\tau +d, \end{array}\right.$$

where the lumped disturbance is defined as

(15) $$d=\tau_{d}-\Delta J\dot{\omega }-\omega^{\times }\Delta J\omega .$$

**Assumption** **1.** *The receiver aircraft operates away from the Euler-angle singularity, i.e., θ≠±π/2.*

**Assumption** **2.** *The lumped disturbance d, including the tanker-wake-induced moment, atmospheric gust disturbance, aerodynamic uncertainty, and inertia perturbation, and its first derivative are assumed to be bounded.*

Aerial-refueling studies have considered tanker-wake disturbances, atmospheric gusts, and aircraft parameter variations in receiver-aircraft modeling and control [1,19,21]. Accordingly, Assumption 2 is adopted for the smooth disturbance profiles within the bounded operating region considered in this study. The above formulation describes the attitude dynamics of the receiver aircraft operating in the tanker wake during aerial refueling. Since the present work focuses on disturbance-rejection attitude stabilization rather than relative-position guidance or docking control, the tanker–receiver relative translational dynamics are not explicitly included. In the subsequent controller design, the virtual torque command τ is assumed to be realizable through the aerodynamic control surfaces.

## 3. Prescribed-Performance ADP Attitude Control Scheme

### 3.1. Finite-Time Prescribed Performance Function

The boundary is generated from a finite-time scalar dynamics rather than postulated directly. For the *i*th attitude channel, let $s_{\rho i}=\varrho_{i}-\varrho_{i\infty }$ and prescribe

(16) $${\dot{s}}_{\rho i}=-c_{\rho i}s_{\rho i}^{q_{\rho i}},\;s_{\rho i}\left(0\right)=\varrho_{i0}-\varrho_{i\infty },$$

where $c_{\rho i}>0$, $0<q_{\rho i}<1$, and $\varrho_{i0}>\varrho_{i\infty }>0$.

**Theorem** **1.** *The solution of *(16)*, continued by zero after its first hitting time, produces the nonincreasing performance envelope*

(17) $$\varrho_{i}\left(t\right)=\varrho_{i\infty }+{\left[{\left(\varrho_{i0}-\varrho_{i\infty }\right)}^{1-q_{\rho i}}-\left(1-q_{\rho i}\right)c_{\rho i}t\right]}_{+}^{\frac{1}{1-q_{\rho i}}},$$

*where ${\left[a\right]}_{+}=\max\left\{a,0\right\}$. The envelope reaches $\varrho_{i\infty }$ at*

(18) $$T_{\rho i}=\frac{{\left(\varrho_{i0}-\varrho_{i\infty }\right)}^{1-q_{\rho i}}}{\left(1-q_{\rho i}\right)c_{\rho i}}.$$

**Proof.**  While $s_{\rho i}>0$, separation of variables in (16) gives

$$s_{\rho i}^{1-q_{\rho i}}\left(t\right)=s_{\rho i}^{1-q_{\rho i}}\left(0\right)-\left(1-q_{\rho i}\right)c_{\rho i}t.$$

The right-hand side vanishes at $T_{\rho i}$ in (18). Consequently, $s_{\rho i}$ is positive and strictly decreasing on $\left[0,T_{\rho i}\right)$ and is set equal to zero thereafter. Adding the constant $\varrho_{i\infty }$ establishes (17) and the claimed boundary properties. □

Collect the three boundaries in $\varrho =\mathrm{col}(\varrho_{1},\varrho_{2},\varrho_{3})$. The normalized attitude is $\chi_{i}=\Theta_{i}/\varrho_{i}$, and the constraint $|\chi_{i}|\;<\;1$ is removed through the inverse-hyperbolic-tangent map

(19) $$\zeta_{i}=\mathrm{artanh}\left(\chi_{i}\right)=\frac{1}{2}\ln\;\left(\frac{1+\Theta_{i}/\varrho_{i}}{1-\Theta_{i}/\varrho_{i}}\right),\;i=1,2,3.$$

Thus, boundedness of $\zeta =\mathrm{col}(\zeta_{1},\zeta_{2},\zeta_{3})$ implies $|\Theta_{i}\left(t\right)|<\varrho_{i}\left(t\right)$ for every channel. Direct differentiation of (19) yields

(20) $$\dot{\zeta }=D_{\zeta }\left(\dot{\Theta }-\Lambda_{\varrho }\Theta \right),$$

where

(21) $$D_{\zeta }={\mathrm{diag}}_{i=1}^{3}\left\{\frac{\varrho_{i}}{\varrho_{i}^{2}-\Theta_{i}^{2}}\right\},\;\Lambda_{\varrho }={\mathrm{diag}}_{i=1}^{3}\left\{\frac{{\dot{\varrho }}_{i}}{\varrho_{i}}\right\}.$$

With $x=\mathrm{col}\left(\zeta ,\omega \right)\in R^{6}$, Equations (14) and (20) take the control-affine form

(22) $$\dot{x}=f\left(x,t\right)+g\left(x\right)\left(\tau +d\right),$$

where

(23) $$f=\mathrm{col}\;\left(D_{\zeta }\left[\Pi \left(\Theta \right)\omega -\Lambda_{\varrho }\Theta \right],-J_{0}^{-1}\left(\omega \times J_{0}\omega \right)\right),\;g=\mathrm{col}\left(0_{3\times 3},J_{0}^{-1}\right).$$

The arguments of f and g are suppressed below whenever they are clear. This representation separates the performance transformation from the observer and learning design used in the following subsections.

### 3.2. Finite-Time Disturbance Observer

All unknown moments in (22), including the tanker wake, gusts, aerodynamic mismatch, and inertia perturbation, are grouped in d. The observer is constructed for the state-space disturbance $w=g\left(x\right)d\in R^{6}$; the physical moment is recovered only after w has been estimated.

Introduce the stable filter

(24) $${\dot{x}}_{f}=-k_{o}x_{f}+g\left(x\right)\tau ,\;k_{o}>0,$$

and define the measurable residual $r=x-x_{f}$. Combining (22) and (24) gives the identity

(25) $$w=\dot{r}+k_{o}r-k_{o}x-f\left(x,t\right).$$

Accordingly, disturbance reconstruction reduces to obtaining a finite-time estimate of $\dot{r}$ and a convergent copy of r.

**Assumption** **3.** *The residual is twice differentiable and satisfies $∥\ddot{r}\left(t\right)∥\leq L_{r}$ for a known constant $L_{r}>0$.*

The justification for this assumption is provided in Appendix A. The derivative channel is generated by the componentwise second-order sliding-mode differentiator

(26) $$\begin{array}{cc} {\dot{\nu }}_{1} & =\nu_{2}-K_{d1}{\mathrm{sig}}^{1/2}\left(e_{\nu }\right),\\ {\dot{\nu }}_{2} & =-K_{d2}\mathrm{sig}\left(e_{\nu }\right), \end{array}\;e_{\nu }=\nu_{1}-r,$$

where $K_{d1},K_{d2}\in R^{6\times 6}$ are positive-definite diagonal matrices. Denote $\hat{\dot{r}}=\nu_{2}$ and $\epsilon_{\nu }=\hat{\dot{r}}-\dot{r}$. Under Assumption 3, standard homogeneity arguments ensure the existence of a finite $T_{\nu }$ such that $\epsilon_{\nu }\left(t\right)=0$ for all $t\geq T_{\nu }$ in the ideal noise-free case.

**Theorem** **2.** *Let $1/2<q_{o}<1$ and $k_{r}>0$. Consider*

(27) $$\begin{array}{cc} \dot{\hat{r}} & =\hat{\dot{r}}-k_{o}\tilde{r}-k_{r}{\mathrm{sig}}^{2q_{o}-1}\left(\tilde{r}\right), \end{array}$$

(28) $$\begin{array}{cc} \hat{w} & =\hat{\dot{r}}+k_{o}\hat{r}-k_{o}x-f\left(x,t\right), \end{array}$$

*where $\tilde{r}=\hat{r}-r$. Then $\hat{w}$ coincides with w after a finite transient. Because g has full column rank, the corresponding moment estimate is $\hat{d}=g^{†}\hat{w}$.*

**Proof.**  Subtracting (25) from (28) first gives

(29) $$\tilde{w}:=\hat{w}-w=k_{o}\tilde{r}+\epsilon_{\nu }.$$

It therefore suffices to establish finite-time convergence of $\tilde{r}$. Equations (27) and (26) yield

(30) $$\dot{\tilde{r}}=-k_{o}\tilde{r}-k_{r}{\mathrm{sig}}^{2q_{o}-1}\left(\tilde{r}\right)+\epsilon_{\nu }.$$

After $T_{\nu }$, the last term is zero. For $V_{o}=\frac{1}{2}{\tilde{r}}^{T}\tilde{r}$,

(31) $$\begin{array}{cc} {\dot{V}}_{o} & =-k_{o}∥\tilde{r}{∥}^{2}-k_{r}\sum_{j=1}^{6}{\left|{\tilde{r}}_{j}\right|}^{2q_{o}} \end{array}$$

(32) $$\begin{array}{cc} \; & \leq -2^{q_{o}}k_{r}V_{o}^{q_{o}}. \end{array}$$

The finite-time comparison lemma then gives

(33) $$T_{o}\leq \frac{V_{o}^{1-q_{o}}\left(T_{\nu }\right)}{2^{q_{o}}k_{r}\left(1-q_{o}\right)}.$$

Hence $\tilde{r}=0$ for $t\geq T_{\nu }+T_{o}$. Equation (29) simultaneously gives $\tilde{w}=0$, and the left inverse of g recovers $\hat{d}=d$. The total reconstruction time is $T_{2}=T_{\nu }+T_{o}$. □

### 3.3. ADP-Based Auxiliary Policy and Practical Stabilization

Once $t\geq T_{2}=T_{\nu }+T_{o}$, the estimate satisfies $\hat{d}=d$. It is therefore convenient to separate the commanded moment into an auxiliary policy u and disturbance cancellation,

(34) $$\tau =u-\hat{d},\;\dot{x}=f\left(x\right)+g\left(x\right)u.$$

The autonomous representation applies after all performance envelopes have reached their constant terminal values.

For an admissible auxiliary policy, define the infinite-horizon return

(35) $$J\left(x_{0},u\right)=\int_{0}^{\infty }l\left(x\left(t\right),u\left(t\right)\right)\;dt,\;l\left(x,u\right)=x^{T}Qx+u^{T}Ru,$$

where Q≻0 and R≻0. If $J^{*}$ is differentiable, dynamic programming gives

(36) $$0=\min_{u}\left\{l\left(x,u\right)+\nabla J^{*T}\left[f+gu\right]\right\}.$$

Stationarity with respect to u produces

(37) $$u^{*}=-\frac{1}{2}R^{-1}g^{T}\nabla J^{*}\left(x\right).$$

The implementable policy is introduced before its ideal approximation. Let $\varphi :\Omega \to R^{l}$ be a continuously differentiable basis vector satisfying φ(0)=0, and set

(38) $$\hat{J}\left(x\right)=\varphi^{T}\left(x\right){\hat{w}}_{c},\;\hat{u}\left(x\right)=-\frac{1}{2}R^{-1}g^{T}\nabla \varphi^{T}\left(x\right){\hat{w}}_{c}.$$

For analysis, write the value function on the compact operating set as

(39) $$J^{*}\left(x\right)=\varphi^{T}\left(x\right)w_{c}^{*}+\varepsilon_{c}\left(x\right),$$

and define ${\tilde{w}}_{c}=w_{c}^{*}-{\hat{w}}_{c}$. The preceding equations then imply the analysis identity

(40) $$\hat{u}=u^{*}+\frac{1}{2}R^{-1}g^{T}\left[\nabla \varepsilon_{c}+\nabla \varphi^{T}{\tilde{w}}_{c}\right].$$

**Assumption** **4.** *The optimal value and policy exist on *Ω. Moreover, $\nabla \varepsilon_{c}$, ∇φ, and $w_{c}^{*}$ are uniformly bounded there by positive constants $L_{\varepsilon }$, $L_{\varphi }$, and $L_{w}$, respectively.

The critic weights are updated according to

(41) $${\dot{\hat{w}}}_{c}=\frac{1}{2\gamma_{c}}\nabla \varphi \;gR^{-1}g^{T}x-\frac{l_{c}\left(x\right)}{\gamma_{c}}\sigma_{c}\left(x\right){\hat{w}}_{c},$$

where $\gamma_{c}>0$, $\sigma_{c}\left(x\right)={∥x∥}^{2}/{(∥x∥}^{2}+\delta_{c})+\epsilon_{c}$ with $\delta_{c},\epsilon_{c}>0$, and

(42) $$0<l_{\min}\leq l_{c}\left(x\right)\leq l_{\max}<\infty ,\;x\in \Omega .$$

For example, $l_{c}\left(x\right)=\lambda_{0}+\lambda_{1}\tanh∥x∥$ satisfies this condition when $\lambda_{0},\lambda_{1}>0$.

The HJB equation in (36) establishes the optimality condition for the critic-based auxiliary policy, which minimizes the approximate Hamiltonian while explicitly penalizing control effort. The critic update law in (41) adaptively updates the value-function parameters, with its regularization term supporting the boundedness result in Theorem 3.

The auxiliary learning policy, nonlinear damping, and disturbance cancellation are combined in the commanded moment

(43) $$\tau =\hat{u}-g^{†}\left(Ax+k_{x}{\mathrm{sig}}^{2q_{x}-1}\left(x\right)\right)-\hat{d},$$

where A≻0, $k_{x}>0$, and $1/2<q_{x}<1$. The fractional-power term is used to strengthen regulation near the equilibrium; no finite-time convergence claim for the complete state is made from this term alone.

The control law in (43) integrates the HJB-based auxiliary policy with nonlinear stabilizing feedback and disturbance compensation. The auxiliary policy minimizes the approximate Hamiltonian to account for control effort, while the additional terms enhance transient regulation and disturbance rejection. This coordinated design achieves a favorable balance among control-effort optimization, transient performance, and robustness. The following result therefore addresses practical boundedness rather than finite-time optimal stabilization.

**Assumption** **5.** *On a compact admissible operating region* Ω containing the origin, let $J^{*}\left(x\right)$ denote the optimal value function associated with the HJB Equation (36), and let $u^{*}\left(x\right)$ be the corresponding optimal policy. Assume that $J^{*}\left(x\right)$ is twice continuously differentiable and satisfies

(44) $${\underline{c}}_{s}{∥x∥}^{2}\leq J^{*}\left(x\right)\leq {\bar{c}}_{s}{∥x∥}^{2},$$

*and*

(45) $$∥\nabla J^{*T}\left(x\right)g\left(x\right)∥\leq L_{J}∥x∥,$$

*where ${\underline{c}}_{s}$, ${\bar{c}}_{s}$, and $L_{J}$ are positive constants. For the control law in *(43), define the effective input mismatch during the autonomous phase as $\Delta_{c}:=\hat{u}-u^{*}-g^{†}\left(Ax+k_{x}{\mathrm{sig}}^{2q_{x}-1}\left(x\right)\right)+d-\hat{d}.$

**Theorem** **3.** *Suppose Assumption 5 and the bounded-approximation conditions stated above hold. Then the critic weights generated by *(41)* are uniformly ultimately bounded. If $∥\Delta_{c}\left(t\right)∥\leq {\bar{\Delta }}_{c}$ during the considered autonomous phase, the transformed state is locally uniformly ultimately bounded and approaches the residual set*

(46) $$\Omega_{x}=\left\{x:∥x∥\leq \sqrt{\frac{{\bar{c}}_{s}c_{\Delta }}{{\underline{c}}_{s}c_{s}}}\;{\bar{\Delta }}_{c}\right\}.$$

*where $c_{s}=\lambda_{\min}\left(Q\right)/2$ and $c_{\Delta }=L_{J}^{2}/\left[2\lambda_{\min}\left(Q\right)\right]$, provided the resulting residual set and the selected invariant sublevel set lie within the admissible region.*

**Proof.**  Rewrite the critic update as

(47) $${\dot{\hat{w}}}_{c}=b_{c}\left(x\right)-a_{c}\left(x\right){\hat{w}}_{c},$$

where

(48) $$b_{c}=\frac{1}{2\gamma_{c}}\nabla \varphi \;gR^{-1}g^{T}x,\;a_{c}=\frac{l_{c}\sigma_{c}}{\gamma_{c}}.$$

All factors in $b_{c}$ are bounded on Ω, so a constant ${\bar{b}}_{c}>0$ exists such that $∥b_{c}∥\leq {\bar{b}}_{c}$. Furthermore, (42) and $\sigma_{c}\geq \epsilon_{c}>0$ imply $a_{c}\geq {\underline{a}}_{c}:=l_{\min}\epsilon_{c}/\gamma_{c}>0$. For $V_{w}=\frac{1}{2}{∥{\hat{w}}_{c}∥}^{2}$, Young’s inequality gives

(49) $$\begin{array}{cc} {\dot{V}}_{w}\; & ={\hat{w}}_{c}^{T}b_{c}-a_{c}∥{\hat{w}}_{c}{∥}^{2}\leq -\frac{{\underline{a}}_{c}}{2}{∥{\hat{w}}_{c}∥}^{2}+\frac{{\bar{b}}_{c}^{2}}{2{\underline{a}}_{c}}. \end{array}$$

Thus ${\hat{w}}_{c}$ is UUB. Since the ideal weight $w_{c}^{*}$ is bounded on Ω, the weight error ${\tilde{w}}_{c}$ is also bounded. The policy (38) and the corresponding contribution to $\Delta_{c}$ are consequently bounded on Ω.

The disturbance-observer error remains bounded during its initial transient and vanishes after $T_{2}$ under the ideal observer assumptions established in Theorem 2. Let $T_{p}$ denote the time at which the prescribed-performance envelope reaches its terminal value. For $t\geq T_{p}$, the nominal transformed dynamics are autonomous. Substituting the control law in (43) into (22) gives

(50) $$\begin{array}{cc} \dot{x}= & f\left(x\right)+g\left(x\right)\hat{u}\left(x\right)-g\left(x\right)g^{†}\left(Ax+k_{x}{\mathrm{sig}}^{2q_{x}-1}\left(x\right)\right)+g\left(x\right)\left(d-\hat{d}\right). \end{array}$$

By the definition of $\Delta_{c}$, these dynamics can be rewritten as

(51) $$\dot{x}=f\left(x\right)+g\left(x\right)\left(u^{*}+\Delta_{c}\right).$$

Taking $V_{s}\left(x\right)=J^{*}\left(x\right)$ and using the HJB Equation (36), we obtain

(52) $$\begin{array}{cc} {\dot{V}}_{s} & =\nabla J^{*T}\left[f+g(u^{*}+\Delta_{c})\right]\\ \; & =-x^{T}Qx-u^{*T}Ru^{*}+\nabla J^{*T}g\Delta_{c}\\ \; & \leq -\lambda_{\min}{\left(Q\right)∥x∥}^{2}+L_{J}∥x∥∥\Delta_{c}∥. \end{array}$$

Applying Young’s inequality yields

(53) $${\dot{V}}_{s}\leq -c_{s}{∥x∥}^{2}+c_{\Delta }{∥\Delta_{c}∥}^{2},$$

where $c_{s}=\lambda_{\min}\left(Q\right)/2$ and $c_{\Delta }=L_{J}^{2}/\left[2\lambda_{\min}\left(Q\right)\right]$. Thus, the ISS inequality follows from the HJB identity and the local gradient bound in Assumption 5.

For the forward-invariance check, choose a compact sublevel set $\Omega_{\rho }=\left\{x:V_{s}\left(x\right)\leq \rho \right\}$ contained in Ω. On its boundary, using (44) and (53) gives

(54) $${\dot{V}}_{s}\leq -\frac{c_{s}}{{\bar{c}}_{s}}\rho +c_{\Delta }{\bar{\Delta }}_{c}^{2}<0,$$

provided that $\rho >\frac{{\bar{c}}_{s}c_{\Delta }}{c_{s}}{\bar{\Delta }}_{c}^{2}.$ If such a sublevel set can be selected within the admissible region, the boundary condition ensures its forward invariance.

Consequently, for bounded $\Delta_{c}$, the transformed state is locally uniformly ultimately bounded and approaches the residual set in (46). The finite-time convergence properties established in Theorems 1 and 2 apply to the prescribed-performance envelope and disturbance reconstruction, respectively, whereas Theorem 3 establishes local uniform ultimate boundedness of the transformed closed-loop state. □

**Figure 2:**
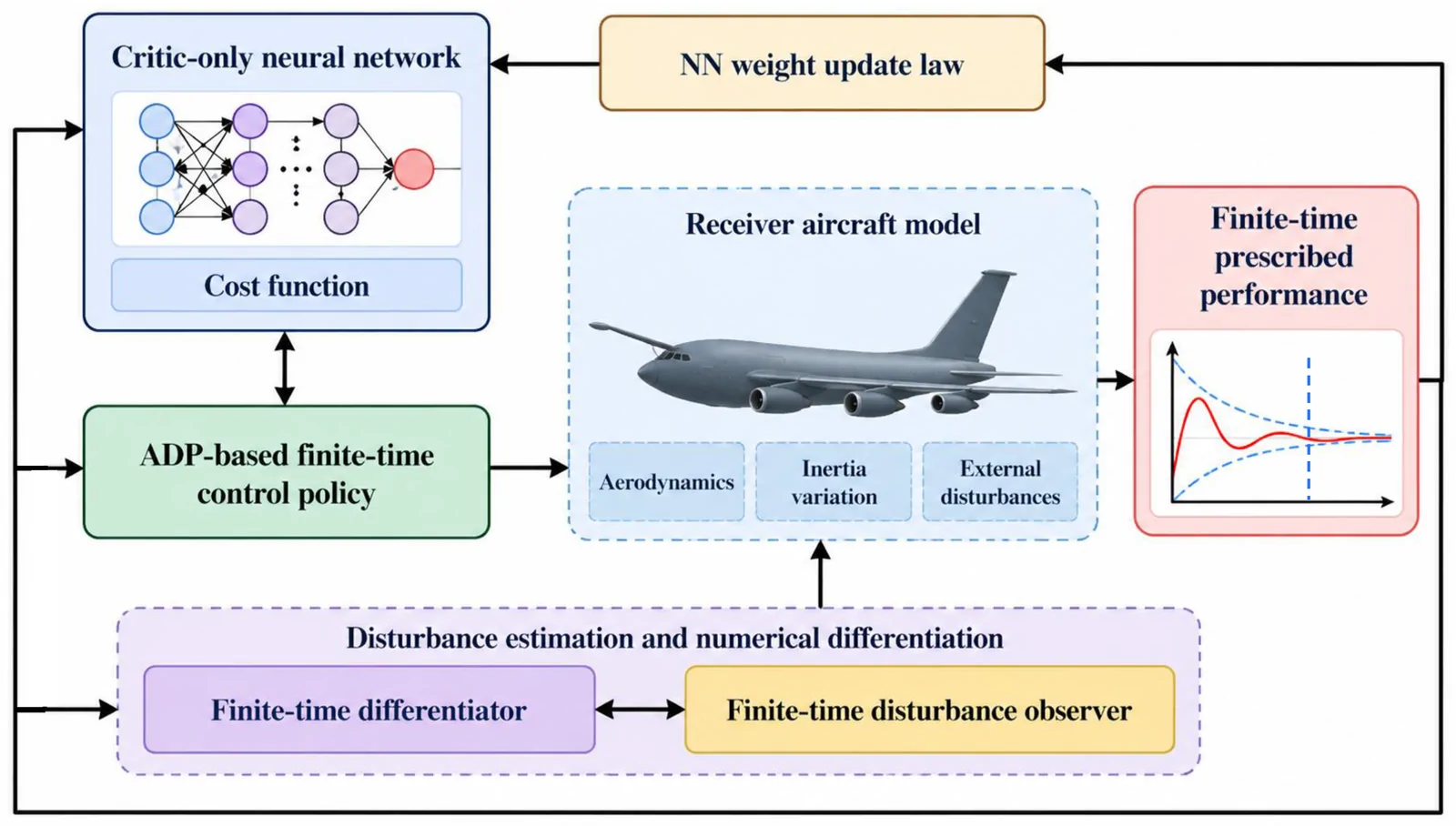
Overall architecture of the proposed prescribed-performance ADP control scheme for the receiver aircraft.

The overall architecture of the proposed prescribed-performance ADP attitude control scheme for the aerial-refueling receiver aircraft is illustrated in Figure 2. The selection of controller and observer parameters plays an important role in achieving a desirable trade-off among convergence speed, disturbance rejection capability, robustness, and control effort. Accordingly, the following remark provides the gain tuning principles.

**Remark** **1.** *The selection of the observer and controller gains involves a trade-off between convergence speed, disturbance rejection capability, and control effort. Specifically, the gain $k_{o}$ in the auxiliary system determines the decay rate of the linear error dynamics of the disturbance observer. A larger $k_{o}$ improves the transient estimation performance but may increase sensitivity to measurement noise.*

*The differentiator gains $K_{d1}$ and $K_{d2}$ are selected based on the estimated residual-derivative bound. Specifically, $K_{d2}$ is chosen to dominate the residual acceleration bound, while $K_{d1}$ is selected to satisfy the finite-time differentiation conditions. The gains are further tuned to balance convergence speed, estimation accuracy, and noise sensitivity, as illustrated in subsequent simulation section.*

*The nonlinear observer gain $k_{r}$ and the exponent $q_{o}$ jointly determine the finite-time convergence property of the disturbance estimation error. A larger $k_{r}$ accelerates the convergence process, while $q_{o}\in \left(0.5,1\right)$ adjusts the convergence behavior near the origin. Smaller values of $q_{o}$ provide faster convergence when the estimation error is large but may introduce higher sensitivity to noise.*

*For the nonlinear damping term, the gain $k_{x}$ controls the strength of feedback applied to the transformed state. Increasing $k_{x}$ can improve transient regulation, while excessively large values may increase control effort and cause actuator saturation. The exponent $q_{x}\in \left(0.5,1\right)$ adjusts the local shape of the fractional-power feedback: a smaller value strengthens its action near the equilibrium, whereas a larger value generally provides smoother control action.*

*Therefore, the gains should be selected sequentially according to the following principle: first choose $k_{o},K_{d1},K_{d2},k_{r},q_{o}$ to ensure sufficient disturbance estimation performance; then select $k_{x},q_{x}$ to obtain the desired practical regulation performance while respecting actuator constraints. In practical implementation, the gains are tuned to achieve a balance between convergence speed, robustness, and control input magnitude.*

## 4. Simulation and Hardware-in-the-Loop Validation

To verify the effectiveness of the proposed prescribed-performance ADP control scheme, numerical simulations and hardware-in-the-loop (HIL) experiments are conducted based on a fixed-wing aircraft platform. The considered aircraft model adopts the aerodynamic configuration introduced in the previous section, where the control inputs are generated by the aerodynamic control surfaces. The simulation results are used to evaluate the tracking performance, disturbance rejection capability, practical regulation accuracy, and control-effort characteristics of the proposed method. Finite-time convergence is assessed only for the performance boundary and disturbance-observation stage.

The main physical parameters of the fixed-wing aircraft are given as follows. The aircraft mass is selected as m=6kg, and the nominal inertia matrix is given by

$$J_{0}=\left[\begin{array}{ccc} 0.25 & 0 & 0\\ 0 & 0.32 & 0\\ 0 & 0 & 0.55 \end{array}\right]\mathrm{kg}\cdot m^{2}.$$

The aerodynamic reference parameters are selected as $S_{\mathrm{ref}}=0.45\;m^{2},\;\bar{b}=1.8\;m,$$\;\bar{c}=0.25\;m$, where $S_{\mathrm{ref}}$, $\bar{b}$, and $\bar{c}$ denote the reference area, wing span, and mean aerodynamic chord, respectively.

### 4.1. Simulation Results of Proposed Disturbance Observer

To independently evaluate the disturbance reconstruction performance of the proposed finite-time disturbance observer, numerical simulations are first conducted before examining the complete closed-loop control system. The objective of this subsection is to verify the finite-time convergence property, reconstruction accuracy, and tracking capability of the proposed observer in the presence of continuously varying aerodynamic disturbances. In accordance with Assumption 2, the injected disturbances are selected as smooth time-varying signals with bounded derivatives.

To test the observer under simultaneous multi-axis disturbances, the lumped disturbance torque is imposed as

(55) $$d\left(t\right)=\left[\begin{array}{c} 0.12\sin(0.3t)+0.04\cos(0.6t)\\ 0.10\cos(0.25t)+0.03\sin(0.5t)\\ 0.08\sin(0.4t)+0.04\cos(0.2t) \end{array}\right]\;N\cdot m.$$

The above disturbance contains components with different amplitudes and frequencies and is used to represent the combined effects of aerodynamic coefficient uncertainties, gust-induced moments, and other unmodeled perturbations. During aerial refueling, the receiver aircraft is subject to time-varying disturbances arising from tanker-wake effects, atmospheric gusts, and aerodynamic uncertainties. In this study, sinusoidal signals are adopted to represent the resulting time-varying lumped disturbances. By adjusting their amplitudes and frequencies, different disturbance intensities and temporal variations can be systematically evaluated. This choice provides a controlled and reproducible means of assessing disturbance reconstruction and attitude regulation under the bounded disturbance conditions considered in this study. The use of sinusoidal signals for observer performance evaluation is also consistent with previous studies [20]. The prescribed disturbance magnitudes and directions define the test conditions, with the magnitude determining the disturbance intensity and the direction determining its effect on the coupled receiver-aircraft dynamics.

The gain matrices of the second-order sliding-mode finite-time differentiator are chosen as $K_{d1}=1I_{6}$ and $K_{d2}=25I_{6}$, where the gains are selected sufficiently large with respect to the prescribed upper bound of $|\ddot{r}|$ to ensure finite-time differentiation in the ideal noise-free case. The nonlinear disturbance observer parameters are selected as $k_{r}=1$ and $k_{o}=1$. The initial states of the auxiliary system and the differentiator are set to $x_{f}\left(0\right)=0_{6},\nu_{1}\left(0\right)=0_{6},\nu_{2}\left(0\right)=0_{6}$. The initial disturbance estimate is taken as $\hat{d}\left(0\right)=0_{3}$.

**Figure 3:**
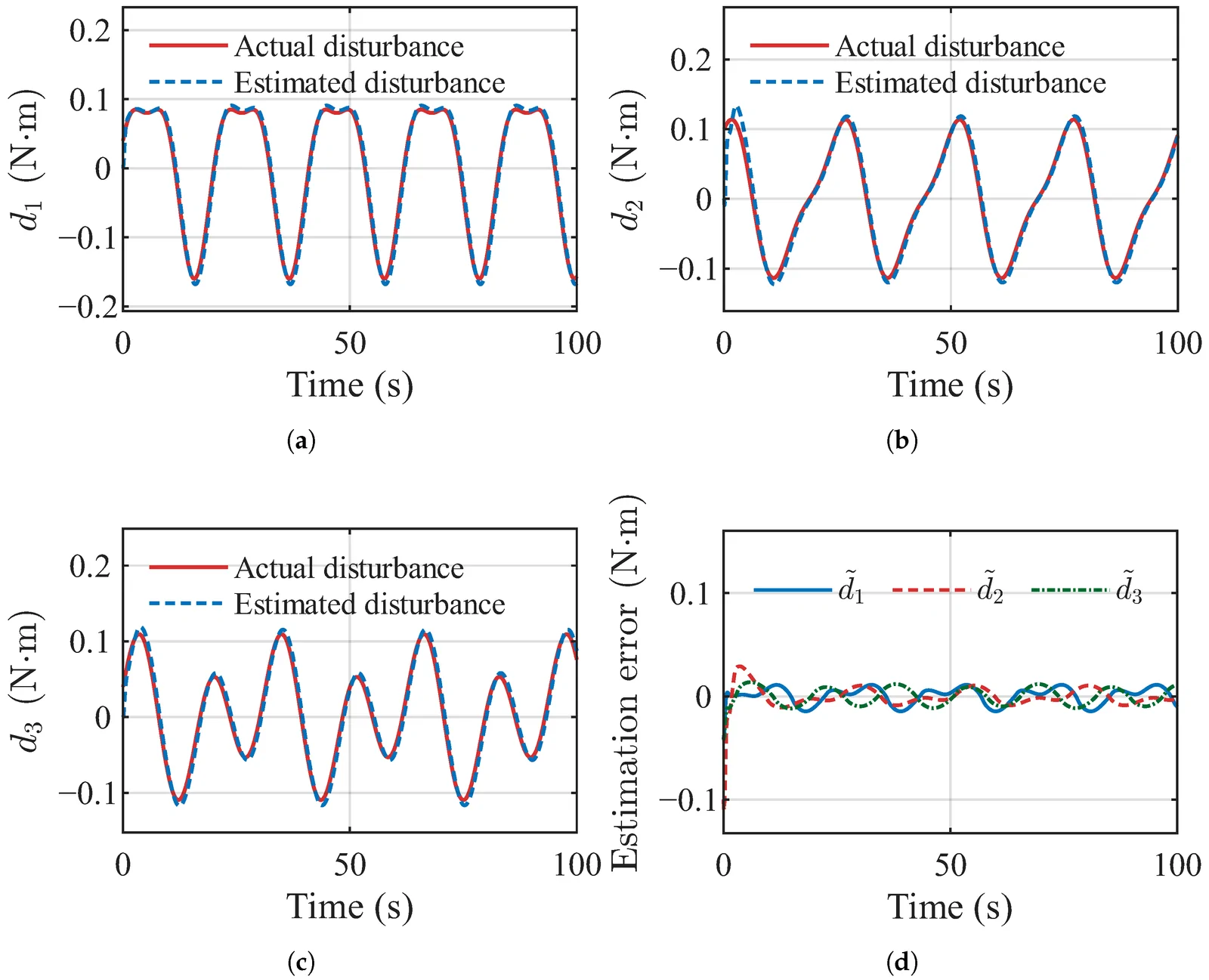
Disturbance estimation performance of the proposed observer: (**a**) Actual and estimated disturbance in the first rotational channel, $d_{1}$ and ${\hat{d}}_{1}$. (**b**) Actual and estimated disturbance in the second rotational channel, $d_{2}$ and ${\hat{d}}_{2}$. (**c**) Actual and estimated disturbance in the third rotational channel, $d_{3}$ and ${\hat{d}}_{3}$. (**d**) Estimation errors of the three disturbance components, ${\tilde{d}}_{1}$, ${\tilde{d}}_{2}$, and ${\tilde{d}}_{3}$.

The simulation results are given in Figure 3.

Figure 3 presents the disturbance reconstruction performance of the proposed observer. As shown in Figure 3a–c, the estimated disturbances closely track the actual disturbances in all three rotational channels, despite the differences in disturbance amplitudes and variation frequencies. The estimated curves almost overlap with the corresponding actual disturbance signals over the entire simulation interval, demonstrating the strong tracking capability of the proposed observer for time-varying disturbances.

The corresponding estimation errors are further illustrated in Figure 3d. Although relatively larger estimation errors occur during the initial transient stage, all three error components rapidly decrease toward a small neighborhood of zero and subsequently remain at a very low level. Compared with the amplitudes of the injected disturbances, the residual estimation errors are considerably smaller, indicating that the proposed observer achieves high-accuracy disturbance reconstruction. Furthermore, no obvious phase lag or amplitude distortion can be observed between the actual and estimated disturbance signals. These results verify that the proposed disturbance observer can accurately reconstruct multi-axis, continuously varying lumped disturbances and provide reliable disturbance information for the subsequent compensation control.

**Figure 4:**
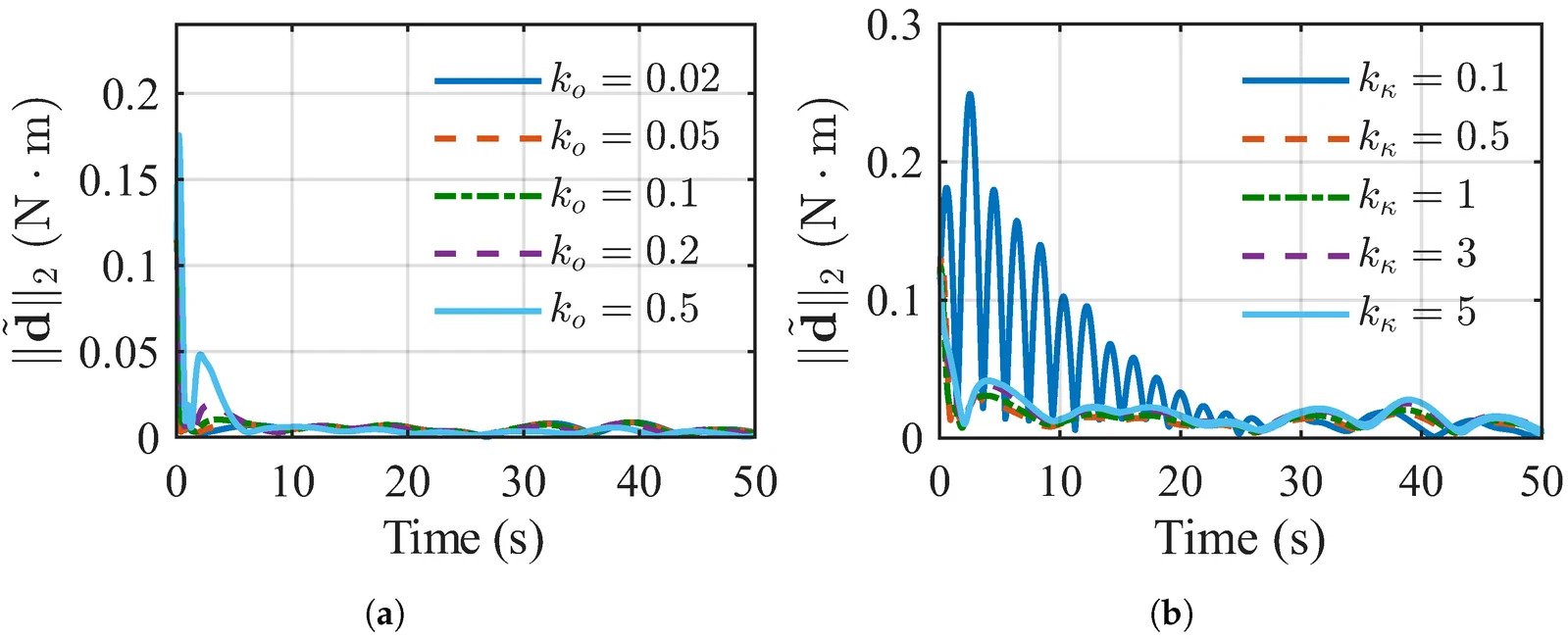
Parameter sensitivity analysis of the proposed disturbance observer: (**a**) influence of the auxiliary-system gain $k_{o}$ on the disturbance estimation error norm; (**b**) influence of the differentiator gain scaling factor κ on the disturbance estimation error norm, where $K_{d1}=k_{\kappa }K_{d1,0}$ and $K_{d2}=k_{\kappa }K_{d2,0}$, and $K_{d1,0}$ and $K_{d2,0}$ denote the baseline differentiator gains specified previously.

Figure 4 shows the influence of the observer parameters on the disturbance reconstruction performance. As illustrated in Figure 4a, a small $k_{o}$ leads to a larger transient estimation error, whereas a moderate increase in $k_{o}$ improves the transient response. However, excessively large values provide no further improvement, indicating that $k_{o}$ should be selected to balance convergence speed and estimation smoothness. Figure 4b presents the influence of the differentiator gain scaling factor κ, where $K_{d1}=k_{\kappa }K_{d1,0}$ and $K_{d2}=k_{\kappa }K_{d2,0}$, with $K_{d1,0}$ and $K_{d2,0}$ being the baseline gains defined previously. A small $k_{\kappa }$ results in slower convergence and larger oscillations, while a moderate value significantly improves the estimation performance. Further increasing $k_{\kappa }$ yields limited improvement and may increase transient oscillations. These results demonstrate that the proposed observer maintains good reconstruction performance over a reasonable range of gain variations.

### 4.2. Experimental Results of the Proposed Prescribed-Performance ADP Controller

**Figure 5:**
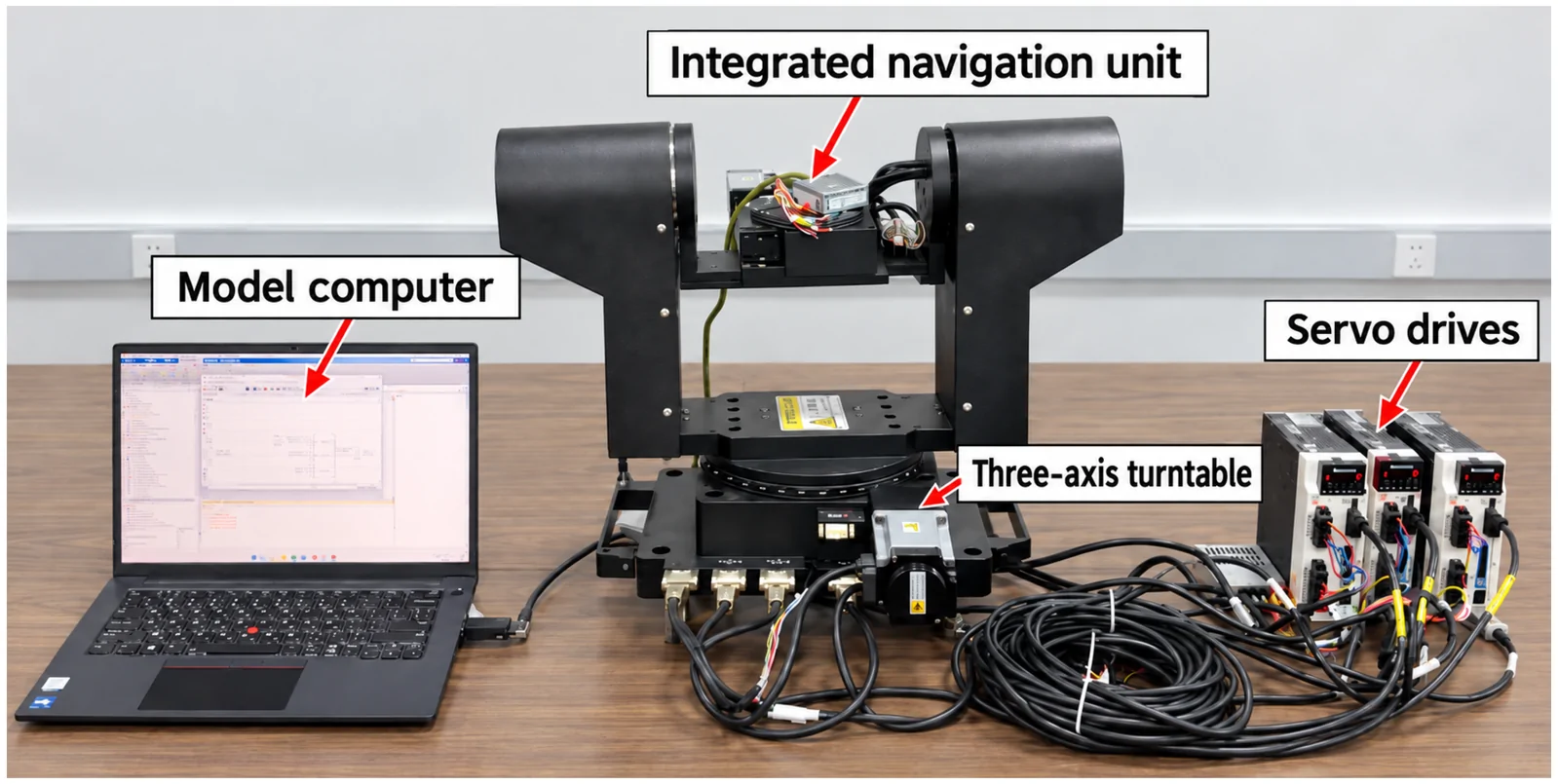
Hardware-in-the-loop experimental platform for receiver-aircraft attitude control.

To further verify the feasibility of the proposed method in practical applications, a laboratory-level hardware-in-the-loop (HIL) platform was established, as shown in Figure 5. The platform consists of a control simulation computer, a WMD3 three-axis rotary stage (Shenzhen Wanming Technology Co., Ltd., Shenzhen, China), an HC-GS/OEM integrated navigation system, and a visual simulation computer. The control simulation computer runs MATLAB R2025b (MathWorks, Natick, MA, USA) to execute the model simulation and control algorithm at a sampling period of $T_{s}=0.05\;s$ (20 Hz). The computed control commands are transmitted to the rotary-stage motor drivers via an RS-485 bus, while the integrated navigation system measures the attitude angles and angular rates and feeds them back to the computer, thereby closing the control loop. Simulation data are transmitted via UDP to the visual simulation computer for monitoring the simulated flight states. Recent research on AHRS-based UAV navigation likewise highlights the importance of real-time attitude information derived from inertial sensing [39]. The controller, disturbance observer, and measurement feedback are implemented digitally. No additional low-pass filtering or continuous-time approximation is introduced specifically to suppress discrete-time chattering.

The HIL experiments validate the real-time implementation and attitude regulation performance of the proposed controller using a three-axis attitude platform. The receiver-aircraft aerodynamic dynamics and disturbance effects are simulated numerically, rather than physically reproduced by the experimental platform. Therefore, the HIL results demonstrate the implementation feasibility of the proposed control framework under the tested conditions, rather than a full reproduction of realistic aerial-refueling operations.

The initial attitude of the receiver aircraft is selected as $\Theta \left(0\right)={\left[5^{\circ },-3^{\circ },8^{\circ }\right]}^{T}$, with the initial angular velocity $\omega \left(0\right)={\left[0,0,0\right]}^{T}\;\mathrm{rad}/s$. To emulate the disturbance environment encountered by the receiver aircraft in the wake-affected region during aerial refueling, the external disturbance moment is constructed as

(56) $$\tau_{d}\left(t\right)=\tau_{\mathrm{wake}}\left(t\right)+\tau_{\mathrm{gust}}\left(t\right)+\tau_{\mathrm{aero}}\left(t\right),$$

where the tanker-wake-induced disturbance is selected as

(57) $$\tau_{\mathrm{wake}}\left(t\right)=\left[\begin{array}{c} 0.12\sin(0.8t)+0.05\sin(2.0t)\\ 0.08\cos(0.6t)+0.03\sin(1.5t)\\ 0.10\sin(0.7t+\pi /6)+0.04\cos(1.8t) \end{array}\right]N\cdot m,$$

the atmospheric-gust-induced disturbance is given by

(58) $$\tau_{\mathrm{gust}}\left(t\right)=\left[\begin{array}{c} 0.025\sin(0.25t)+0.015\cos(1.1t)\\ 0.020\sin(0.30t+0.4)\\ 0.018\cos(0.27t)+0.012\sin(1.3t) \end{array}\right]N\cdot m,$$

and the bounded aerodynamic uncertainty is modeled as

(59) $$\tau_{\mathrm{aero}}\left(t\right)=\left[\begin{array}{c} 0.020\sin(0.15t)\\ 0.015\cos(0.12t)\\ 0.018\sin(0.18t+0.3) \end{array}\right]N\cdot m.$$

In addition, a 5% inertia perturbation, $\Delta J=0.05J_{0}$, is introduced to account for parametric uncertainty. The prescribed performance parameters are selected as $\varrho_{0}={\left[15^{\circ },15^{\circ },20^{\circ }\right]}^{T}$, $\varrho_{\infty }={\left[0.5^{\circ },0.5^{\circ },1^{\circ }\right]}^{T}$, and $c_{\rho i}=0.8$, i=1,2,3.

To demonstrate the effectiveness of the proposed method, three controllers are compared under the same simulation conditions: the proposed prescribed-performance finite-time ADP controller (PFTOC), a robust finite-time sliding-mode controller (RFTC), and a classical ADP controller (CADP) constructed following the continuous-time ADP formulation in Ref. [28]. The RFTC is given by

(60) $$\left\{\begin{array}{cc} s & =\omega +\beta \zeta ,\\ \tau_{\mathrm{RFTC}} & =\omega^{\times }J_{0}\omega -J_{0}\left[\beta \omega +k_{x}{\mathrm{sig}}^{q_{x}}\left(s\right)\right]-\hat{d}. \end{array}\right.$$

All three controllers are tested using the same initial conditions, disturbance inputs, and sampling period $T_{s}=0.05\;s$. Their attitude regulation, disturbance rejection, and control performance are then compared.

**Figure 6:**
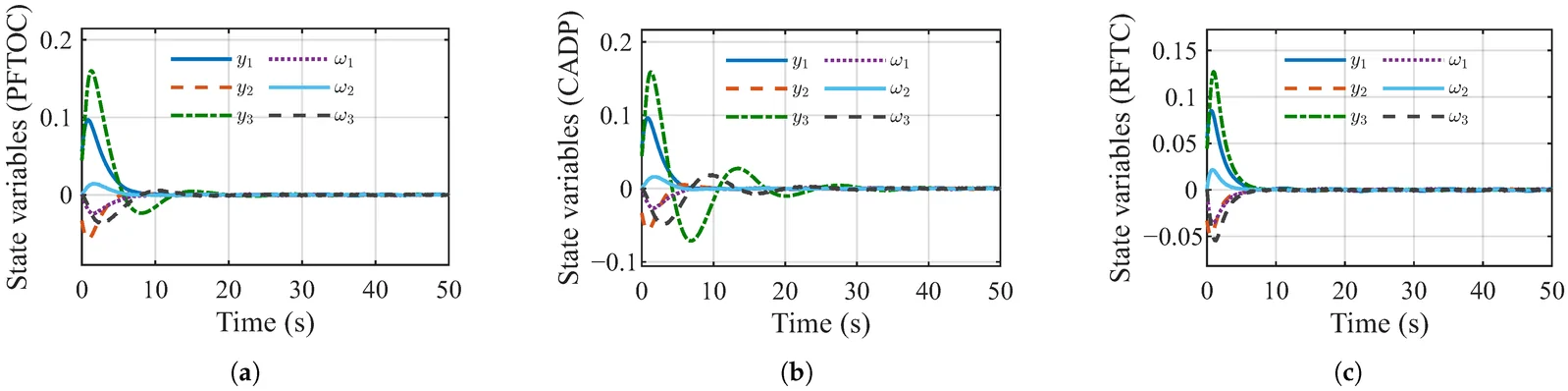
State responses under different control methods: (**a**) PFTOC; (**b**) CADP; (**c**) RFTC.

**Figure 7:**
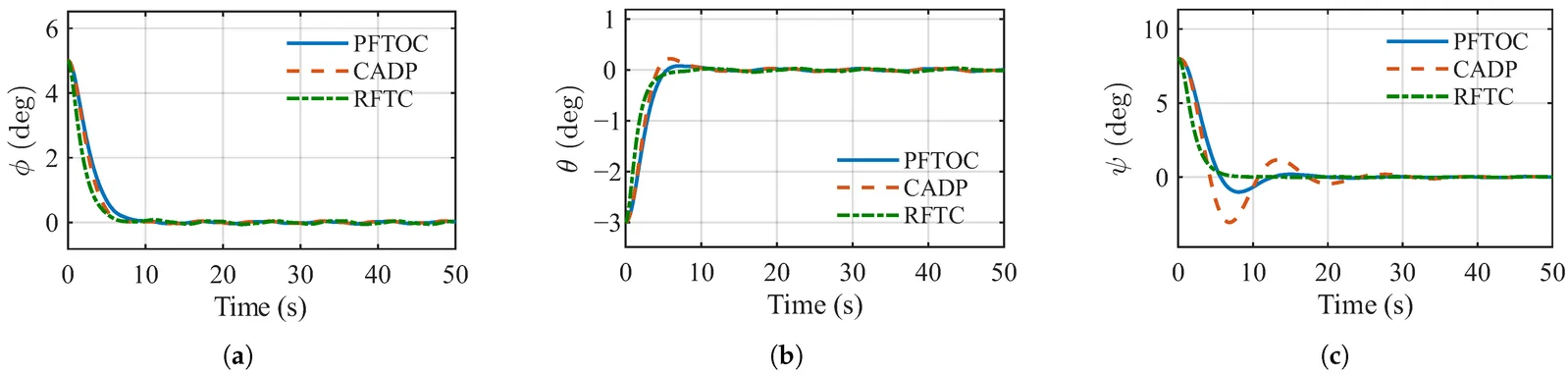
Comparison of the attitude responses under different control methods: (**a**) roll-angle response ϕ; (**b**) pitch-angle response θ; (**c**) yaw-angle response ψ.

**Figure 8:**
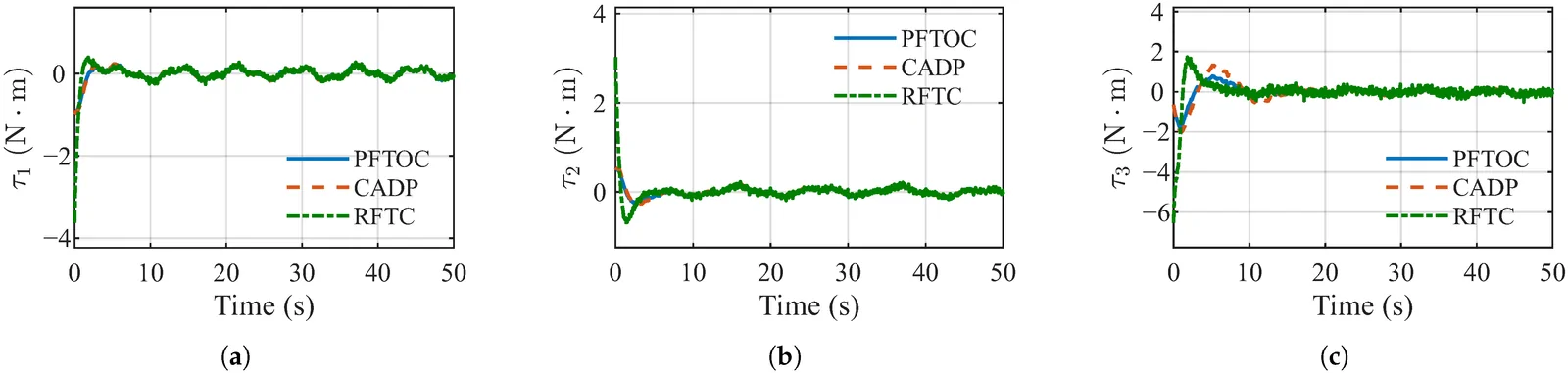
Control-input comparison among PFTOC, CADP, and RFTC: (**a**) rolling control torque $\tau_{1}$; (**b**) pitching control torque $\tau_{2}$; (**c**) yawing control torque $\tau_{3}$.

**Figure 9:**
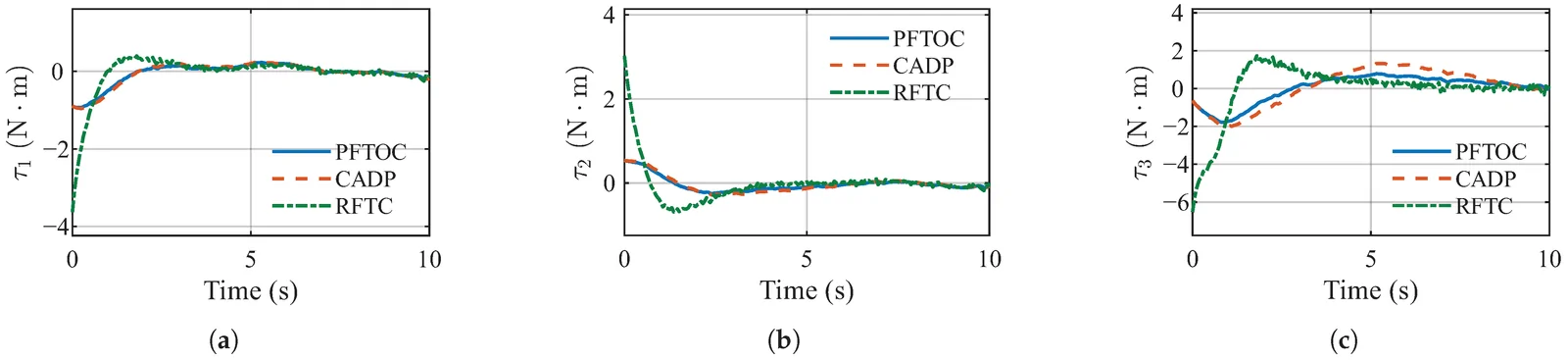
Enlarged control-input comparison among PFTOC, CADP, and RFTC during the initial 0–10s interval: (**a**) rolling control torque $\tau_{1}$; (**b**) pitching control torque $\tau_{2}$; (**c**) yawing control torque $\tau_{3}$.

For a fair comparison, the same finite-time disturbance observer is employed for PFTOC, CADP, and RFTC to estimate and compensate for the lumped disturbance. Owing to the disturbance compensation provided by the observer, all three control schemes are able to stabilize the receiver-aircraft attitude system under the considered disturbance conditions. The comparative results are presented in Figure 6, Figure 7, Figure 8 and Figure 9. As shown in Figure 6, the main differences among the three methods are therefore reflected in their transient responses and control effort. CADP exhibits relatively larger transient oscillations in some state channels, whereas PFTOC provides a smoother convergence process with smaller fluctuations. RFTC also achieves rapid state convergence, but its more aggressive transient response is accompanied by a considerably larger control effort.

The attitude responses in Figure 7 further show that the three methods can regulate the roll, pitch, and yaw angles to the desired equilibrium. Compared with CADP, PFTOC produces smaller transient oscillations, particularly in the yaw channel, and approaches the equilibrium more smoothly. Although RFTC exhibits a fast transient response, this improvement is obtained at the expense of a much stronger control action.

This feature can be clearly observed from Figure 8. The RFTC generates significantly larger initial control torques and noticeable high-frequency fluctuations, especially in the pitching and yawing channels. In contrast, the control inputs generated by PFTOC remain relatively smooth and moderate while maintaining satisfactory attitude convergence. CADP also provides bounded control inputs, but relatively larger transient variations can be observed in some channels.

To further illustrate the contribution of the ADP component, RFTC is included as an ADP-free baseline. While PFTOC incorporates a critic-generated policy to approximate the optimal control action, RFTC relies on finite-time robust feedback. The quantitative results show that PFTOC reduces the integrated control effort from 26.369 to $7.513\;{\left(N\;m\right)}^{2}s$ and the peak resultant control torque from 8.057 to 1.914Nm compared with RFTC. Although RFTC achieves faster convergence and a lower RMS attitude error, PFTOC maintains satisfactory attitude regulation with substantially lower control expenditure. These results highlight the practical benefit of incorporating the ADP policy into the proposed control framework.

### 4.3. Quantitative Analysis

To further quantify the performance of the three controllers, Table 1 summarizes their settling times, RMS attitude errors, maximum overshoots, and control-effort metrics over the 50 s simulation period. PFTOC reduces the settling time from 28.75 s to 16.30 s and the maximum overshoot from 3.060° to 1.008° compared with CADP. It also reduces the integrated control effort by approximately 44.0%. Compared with RFTC, PFTOC reduces the integrated control effort by approximately 71.5% and the peak resultant control torque by 76.2%, although RFTC achieves faster convergence and a lower RMS attitude error. These results demonstrate that PFTOC provides a favorable balance between attitude regulation performance and control expenditure.

## 5. Conclusions

This paper develops a prescribed-performance ADP attitude-control scheme for an aerial-refueling receiver aircraft subject to tanker-wake disturbances and model uncertainties. A finite-time observer reconstructs the lumped disturbance, while a critic-generated auxiliary policy and nonlinear damping term improve attitude regulation and control performance. The analysis shows bounded critic weights and local uniform ultimate boundedness of the closed-loop signals under the stated input-to-state stability condition; it does not assert finite-time convergence of the complete state. Comparative simulations with CADP and RFTC demonstrate that the proposed PFTOC controller achieves smoother transient responses and a favorable balance between stabilization performance and control effort. HIL experiments further verify its real-time implementability.

The current formulation assumes that the virtual control torque can be realized within the admissible actuator range and does not explicitly account for control-surface saturation or deflection-rate constraints. Although the ADP policy minimizes the prescribed control-effort cost, actuator constraint satisfaction is not explicitly guaranteed. Future work will incorporate actuator magnitude and rate constraints into the proposed control framework to further improve its practical applicability. The HIL experiments provide laboratory-level validation of the proposed control framework, accounting for hardware actuation capabilities, measurement errors, and real-time implementation. However, they do not fully reproduce actual flight conditions. Moreover, the sinusoidal disturbances used do not fully capture realistic tanker-wake vortices. Future work will incorporate more realistic wake models and pursue wind-tunnel or flight tests.

## Figures and Tables

**Table 1:** Quantitative performance comparison of PFTOC, CADP, and RFTC.

Performance Metric	PFTOC	CADP	RFTC
Settling time (s)	16.30	28.75	6.35
RMS attitude error (deg)	1.195	1.230	0.919
Maximum overshoot (deg)	1.008	3.060	0.065
Integrated control effort (${\left(N\;m\right)}^{2}s$)	7.513	13.407	26.369
Peak resultant control torque (N m)	1.914	2.113	8.057

Note: The integrated control effort is defined as $J_{u}=\int_{0}^{50}{∥\tau \left(t\right)∥}_{2}^{2}\;dt,$ where $\tau \left(t\right)={\left[\tau_{1}\left(t\right),\tau_{2}\left(t\right),\tau_{3}\left(t\right)\right]}^{T}$.

## Data Availability

The data are contained in the manuscript.

## Abbreviations

The following abbreviations are used in this manuscript:

ADP	Adaptive dynamic programming
ADRC	Active disturbance rejection control
CADP	Classical adaptive dynamic programming controller
ESO	Extended state observer
HIL	Hardware-in-the-loop
HJB	Hamilton–Jacobi–Bellman
LQR	Linear quadratic regulation
MPC	Model predictive control
PID	Proportional–integral–derivative
PFTOC	Prescribed-performance finite-time ADP controller
RFTC	Robust finite-time controller
UAV	Unmanned aerial vehicle
UUB	Uniformly ultimately bounded

## Appendix A. Residual-Derivative Bound and Observer Robustness

### Appendix A.1. Estimation of the Residual-Derivative Bound

From (22) and (24), the residual dynamics satisfy

(A1) $$\dot{r}=f\left(x,t\right)+gd+k_{o}x_{f}.$$

Since g is constant, differentiation yields

(A2) $$\ddot{r}=\frac{\partial f}{\partial x}\dot{x}+\frac{\partial f}{\partial t}+g\dot{d}+k_{o}{\dot{x}}_{f}.$$

On the considered bounded operating region, suppose that the nominal dynamics and their relevant derivatives are uniformly bounded and that the control input and auxiliary-filter state remain bounded. Under Assumption 2, a conservative residual-derivative bound is given by

(A3) $$L_{r}=L_{\dot{f}}+∥g∥L_{\dot{d}}+k_{o}L_{{\dot{x}}_{f}},$$

where $L_{\dot{f}}$, $L_{\dot{d}}$, and $L_{{\dot{x}}_{f}}$ denote upper bounds on $∥\dot{f}∥$, $∥\dot{d}∥$, and $∥{\dot{x}}_{f}∥$, respectively. These bounds can be estimated from the nominal aircraft dynamics, disturbance variation, and auxiliary-filter dynamics over the considered operating region.

### Appendix A.2. Robustness Analysis

Consider a bounded differentiator error $∥\epsilon_{\nu }∥\;\leq \;{\bar{\epsilon }}_{\nu }$. From (30), with $V_{o}=\frac{1}{2}{∥\tilde{r}∥}^{2}$, one obtains

(A4) $${\dot{V}}_{o}\leq -k_{o}∥\tilde{r}{∥}^{2}+{\bar{\epsilon }}_{\nu }∥\tilde{r}∥.$$

Consequently,

(A5) $$\underset{t\to \infty }{\mathrm{lim sup}}∥\tilde{r}\left(t\right)∥\leq \frac{{\bar{\epsilon }}_{\nu }}{k_{o}}.$$

Furthermore, using (29) and $\hat{d}-d=g^{†}\tilde{w}$ gives

(A6) $$\underset{t\to \infty }{\mathrm{lim sup}}∥\hat{d}-d∥\leq 2∥g^{†}∥{\bar{\epsilon }}_{\nu }.$$

Therefore, the disturbance reconstruction error remains ultimately bounded under a bounded effective differentiator error. Under the ideal conditions of Theorem 2, exact finite-time disturbance reconstruction is recovered.

## References

[1] Ren J., Quan Q. (2024). Progress in modeling and control of probe-and-drogue autonomous aerial refueling. Chin. J. Aeronaut..

[2] Bao X., Li Y., Wang Z. (2025). Direct force control technology for longitudinal trajectory of receiver aircraft based on incremental nonlinear dynamic inversion and active disturbance rejection controller. Machines.

[3] Hu J., Wu Y., Su S. (2025). Sliding mode control with power-type barrier function for autonomous aerial refueling based on disturbance observer. ISA Trans..

[4] Ren J., Du J., Xu B., Chen Y., Li Z. (2026). Multi-degree-of-freedom reliable docking control for probe-and-drogue autonomous aerial refueling. Aerosp. Sci. Technol..

[5] Negru S.A., Geragersian P., Petrunin I., Guo W. (2024). Resilient multi-sensor UAV navigation with a hybrid federated fusion architecture. Sensors.

[6] Wu Z., Wang L., Zhang Z., Xiao B., Qu Y. (2026). Practical predefined-time control for stable and safe docking of fixed-wing receiver in aerial refueling. Aerosp. Sci. Technol..

[7] Anjali B.S., Vivek A., Nandagopal J.L. (2016). Simulation and analysis of integral LQR controller for inner control loop design of a fixed wing micro aerial vehicle (MAV). Procedia Technol..

[8] Reinhardt D., Johansen T.A. (2021). Control of fixed-wing UAV attitude and speed based on embedded nonlinear model predictive control. IFAC-PapersOnLine.

[9] Zhuang H., Sun Q., Chen Z., Zeng X. (2021). Back-stepping active disturbance rejection control for attitude control of aircraft systems based on extended state observer. Int. J. Control Autom. Syst..

[10] Wang Z., Hu L., Fei W., Zhou D., Yang D., Ma C., Gong Z., Wu J., Zhang C., Yang Y. (2023). High-performance attitude control design of supersonic tailless aircraft: A cascaded disturbance rejection approach. Aerospace.

[11] Castañeda H., Salas-Peña O.S., de León-Morales J. (2017). Extended observer based on adaptive second order sliding mode control for a fixed wing UAV. ISA Trans..

[12] Cui L., Jin N., Chang S., Zuo Z., Zhao Z. (2022). Fixed-time ESO based fixed-time integral terminal sliding mode controller design for a missile. ISA Trans..

[13] Bianchi D., Di Gennaro S., Di Ferdinando M., Acosta Lùa C. (2023). Robust control of UAV with disturbances and uncertainty estimation. Machines.

[14] Thanh H.L.N.N., Huynh T.T., Vu M.T., Mung N.X., Phi N.N., Hong S.K., Vu T.N.L. (2022). Quadcopter UAVs extended states/disturbance observer-based nonlinear robust backstepping control. Sensors.

[15] Levant A. (1998). Robust exact differentiation via sliding mode technique. Automatica.

[16] Davila J., Fridman L., Levant A. (2005). Second-order sliding-mode observer for mechanical systems. IEEE Trans. Autom. Control.

[17] Hernández-González O., Targui B., Valencia-Palomo G., Guerrero-Sánchez M.E. (2024). Robust cascade observer for a disturbance unmanned aerial vehicle carrying a load under multiple time-varying delays and uncertainties. Int. J. Syst. Sci..

[18] Hernández-González O., Ramírez-Rasgado F., Farza M., Guerrero-Sánchez M.E., Astorga-Zaragoza C.M., M’Saad M., Valencia-Palomo G. (2024). Observer for nonlinear systems with time-varying delays: Application to a two-degrees-of-freedom helicopter. Aerospace.

[19] Wu C., Hui J., Yan J., Guo Y., Xiao B. (2021). Dynamic modeling and faster finite-time attitude stabilization of receiver aircraft for aerial refueling. Nonlinear Dyn..

[20] Wu C., Yan J., Lin H., Wu X., Xiao B. (2021). Fixed-time disturbance observer-based chattering-free sliding mode attitude tracking control of aircraft with sensor noises. Aerosp. Sci. Technol..

[21] Wu C., Yan J., Shen J., Wu X., Xiao B. (2021). Predefined-time attitude stabilization of receiver aircraft in aerial refueling. IEEE Trans. Circuits Syst. II Express Briefs.

[22] Wu C., Yan J., Wu X., Guo Y., Mou P., Xiao B. (2022). Predefined-time sliding manifold-based fixed-time attitude stabilization control of receiver aircraft with measurement noises. Trans. Inst. Meas. Control.

[23] Zhang Z., Li X., Zhang L. (2024). Finite-time attitude control of quadrotor unmanned aerial vehicle with disturbance and actuator saturation. Appl. Sci..

[24] Yang Y., Kim S., Lee K., Leeghim H. (2024). Disturbance robust attitude stabilization of multirotors with control moment gyros. Sensors.

[25] Wang Y., Wang H., Liu Y., Chen J., Wu T., Zheng W. (2022). Modeling and predefined-time anti-disturbance control for the aerial refueling phase of receiver aircraft. Appl. Math. Model..

[26] Zhao W., Qu Y., Yu Z., Zhang Y. (2024). Finite-time guidance and control for uncrewed receiver aircraft in aerial refueling rendezvous. J. Aerosp. Eng..

[27] Hu J., Wu Y., Su S. (2025). Flexible prescribed performance tracking control for receiver UAV with actuator faults and constraints. J. Frankl. Inst..

[28] Vamvoudakis K.G., Lewis F.L. (2010). Online actor–critic algorithm to solve the continuous-time infinite horizon optimal control problem. Automatica.

[29] Modares H., Lewis F.L. (2014). Optimal tracking control of nonlinear partially-unknown constrained-input systems using integral reinforcement learning. Automatica.

[30] Han X., Zheng Z., Liu L., Wang B., Cheng Z., Fan H., Wang Y. (2020). Online policy iteration ADP-based attitude-tracking control for hypersonic vehicles. Aerosp. Sci. Technol..

[31] Zhang C., Zhang G., Dong Q. (2023). Adaptive dynamic programming-based adaptive-gain sliding mode tracking control for fixed-wing unmanned aerial vehicle with disturbances. Int. J. Robust Nonlinear Control.

[32] Yuan Y., Zhou D. (2024). Reinforcement learning for dual-control aircraft six-degree-of-freedom attitude control with system uncertainty. Aerospace.

[33] Huang X., Zhang Y., Liu J., Zhong H., Wang Z., Peng Y. (2023). Error dynamics based dual heuristic dynamic programming for self-learning flight control. Appl. Sci..

[34] Yang S., Yu F., Liu H., Ma H., Zhang H. (2023). Adaptive-dynamic-programming-based robust control for a quadrotor UAV with external disturbances and parameter uncertainties. Appl. Sci..

[35] Liu Y., Dai Y., Li M., Lichen S., He L. (2026). Predefined-time optimal fault-tolerant attitude control for tailless aerial vehicles via the adaptive dynamic programming. Aerosp. Sci. Technol..

[36] Xu J., Wang L., Liu Y., Xue H. (2023). Finite-time prescribed performance optimal attitude control for quadrotor UAV. Appl. Math. Model..

[37] Yang H., Hu Q., Dong H., Zhao X., Li D. (2023). Optimized data-driven prescribed performance attitude control for actuator saturated spacecraft. IEEE/ASME Trans. Mechatron..

[38] An K., Wang Z., Huang W., Liu S. (2024). Performance-prescribed optimal neural control for hypersonic vehicles considering disturbances: An adaptive dynamic programming approach. Aerosp. Sci. Technol..

[39] Lu Y., Li Z., Xiong J., Lv K. (2024). Adaptive UAV navigation method based on AHRS. Sensors.

